# Three‐dimensional printer use in the Australian and New Zealand radiation therapy setting

**DOI:** 10.1002/jmrs.613

**Published:** 2022-09-12

**Authors:** Christine E Albantow, Savannah J Brown

**Affiliations:** ^1^ Crown Princess Mary Cancer Centre, Westmead Hospital Westmead New South Wales Australia; ^2^ Townsville Cancer Centre Douglas Queensland Australia

**Keywords:** 3D bolus, 3D printing, Australia, New Zealand, radiation therapy

## Abstract

**Introduction:**

This cross‐sectional survey aimed to collect data from radiation therapy departments around Australia and New Zealand to establish a baseline of 3D printer and product use.

**Methods:**

Each department in Australia and New Zealand was contacted to determine the most appropriate person to answer the survey. A Microsoft Forms link to the survey was sent to the individual. The survey contained 47 questions in relation to what 3D printing device departments had (if any), how it was being utilised, and what 3D printed products were in use.

**Results:**

A total of 112 departments completed the survey (100% response rate), with 22.3% reporting 3D printer ownership, and thirty‐four departments (30.4%) outsourcing 3D printed products. The primary use of 3D printers was bolus production (60.9%). Public departments represented 84% of printer ownership, while private departments were the greatest users of outsourced 3D printed products (91.4%). 3D Slicer was the most common software used for Digital Imaging and Communications in Medicine (DICOM) file conversion (42.3%), while polylactic acid (PLA) and acrylonitrile butadiene styrene (ABS) were the most common filaments in use, 46% and 14%, respectively.

**Conclusion:**

This research established a baseline for 3D printer and product use within the Australian and New Zealand radiotherapy setting.

## Graphical abstract

This cross‐sectional survey aimed to established a baseline of 3D printer and product use within Australian and New Zealand radiation therapy departments. Survey response rate was 100% with 22.3% of departments reporting ownership of one or more 3D printers, and 34 departments purchasing 3D printed products from an external provider. This research successfully established a baseline for 3D printer and product use within the radiation therapy setting.

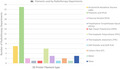

## Introduction

The relatively low cost of production of three‐dimensional (3D) printed products and their versatility suggests 3D printers will soon become standard equipment for radiation therapy (RT) departments, with a large body of evidence supporting their use and applications. Research on 3D printers has been categorised into seven fields: quality assurance phantoms (26.2%), brachytherapy applicators (20.4%), bolus (16.5%), preclinical animal irradiation (9.7%), compensators (6.8%), immobilisation devices (4.9%) and beam modulators (4.9%).^1^ This research has established some basic principles for 3D printed products relevant to dosimetric outcomes within RT. The dosimetric and physical properties of 3D printed objects change between filament suppliers,^2^ printer brands,^3^ print settings such as infill and print direction,^4^ and filament chemical composition. Additionally, it has been reported that polylactic acid (PLA)^5^ and acrylonitrile butadiene styrene (ABS)^6^ are common 3D printer filaments with tissue equivalent radiological properties appropriate for bolus use.

The ability to accurately tailor dose modifiers for improved personalised patient care, create personalised immobilisation equipment and design phantoms and equipment to streamline department workflow are pertinent applications for 3D printing within RT. Despite a large body of research on 3D printing in RT, there are no available statistics concerning rates of department 3D printer ownership, printer applicability or the number of departments outsourcing the production of 3D printed objects from an external supplier. Establishing a baseline of 3D printer use would provide a reference to departments looking to implement a 3D printing program, specifically printer specifications, safety considerations and potential applications. The aim of this study was to establish a baseline of Australian and New Zealand 3D printer ownership and use in the RT environment.

Using the established evidence base to aid in the selection of questions, a survey was constructed for a cross‐sectional review of Australian and New Zealand 3D printer ownership and 3D printed product use within RT. This was the first national and bi‐national review of 3D printed product integration in the RT setting globally. This paper defines 3D printed products as any object created by a 3D printer used within the RT setting including items to modify therapeutic dose, perform quality assurance checks and immobilise objects and people.

## Methods

A cross‐sectional study of Australian and New Zealand RT department 3D printed product use was conducted between 9 August 2020 and 12 September 2020. Responses were de‐identified following public or private characterisation. An institutional ethics waiver was granted for this survey from Townsville Hospital and Health Service Human Research Ethics Committee (Institution reference number: MSG‐20‐29). Simple descriptive statistics summarised the responses. A list of 112 RT departments across Australia and New Zealand was generated from online resources (Appendix A). Departments were contacted by phone to determine whether 3D printed products were in use. Departments using 3D printed products were asked to provide a contact email for the department's most knowledgeable staff on 3D printed products, and an electronic survey link generated through Microsoft Forms™ (Office 365) was sent to the designated individual. Departments who reported no use of 3D printed products or ownership of a 3D printer were also recorded in the Forms™ survey. The survey consisted of 3 multiple‐choice, 16 open‐ended and 28 closed questions (see supplementary information). Study data was collected and managed within Microsoft Forms. Depending upon the answers given to yes or no questions, some of the follow‐up questions were automatically skipped. This altered the number of responses per question and the resulting statistics relevant to each.

## Results

The survey received a 100% response rate, 102 from Australia and 10 from New Zealand. Responses were divided into departments who owned a 3D printer, and who outsourced 3D printed products. Twenty‐five departments (22.3%) reported owning a 3D printer. Thirty‐four departments (30.4%) outsourced 3D printed products. Only one department reported owning a 3D printer as well as outsourcing 3D printed products. The remaining departments did not utilise 3D printed products.

### Distribution of 3D printers

Of the 25 departments with 3D printers, New South Wales reported the highest rate of ownership with nine departments (36%), followed by Victoria with five (20%), Queensland with three (12%), Western Australia with two (8%), Tasmania with two (8%) and South Australia with a single department (4%). The North Island of New Zealand had two departments with 3D printers (8%), and the South Island had one (4%).

**Figure 1:**
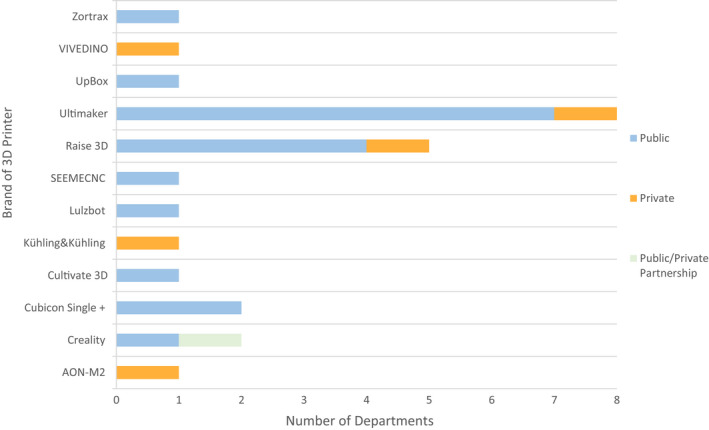
Department‐owned 3D printer brands.

Figure 1 displays the current 3D printer brands owned by each department, with a breakdown of both public and private ownership. Nineteen departments owned a single printer and four departments owned multiple printers. Of the departments with multiple printers, two departments owned ‘four (+)’ printers, one department owned three printers, and one department owned two printers. Two departments reported owning more than one brand of 3D printer; a Raise 3D (Raise 3D Technologies, Inc., Irvine, CA, USA) and Ultimaker (Ultimaker B.V., Utrecht, The Netherlands) and an AON‐M22 (AON3D, Montreal, Canada) and Raise 3D models, respectively. The Ultimaker was the most common 3D printer with 32% of departments with a 3D printer reporting ownership of this brand. Three departments reported they were not clinical with their 3D printers at the time of response. One department reported the printer as not currently functional; the second department did not have a clinical lead for implementation, and the third department used the printer for prototyping only.

### Department usage of 3D products

**Figure 2:**
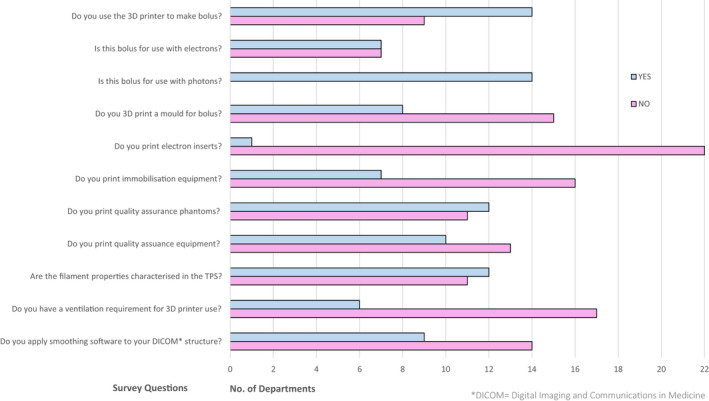
Clinical 3D printed product integration.

The most frequently 3D printed item was bolus, with all departments who printed bolus reporting its use for photons, and half printing bolus for electrons. Figure 2 lists how departments were integrating 3D printed products within their department. Eight departments reported printing a mould for custom patient bolus, with fill for the mould varying and sometimes used in combination with a second item. Eurosil 4 and Dental Wax were the most common fill, both in use by two departments each, followed by paraffin, Eurosil 10, NL‐Tec Super Stuff and PLA. Fourteen departments 3D printed patient‐specific bolus for use with photon treatment and seven departments for use with electron treatment.

**Figure 3:**
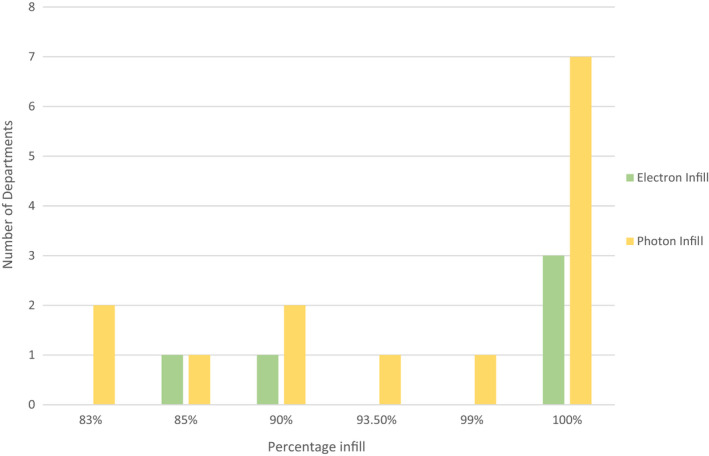
Department infill percentages.

Figure 3 displays the infill percentages used for 3D printed items for photon and electron treatments. The most common infill percentage was 100%, used by seven departments for photon treatments and three departments for electron treatments. One department declined to report the infill percentage used for electron and photon treatments on the grounds of intellectual property. Another department commented that where a 3D printed device was present within a treatment field, but was not acting as bolus, an infill percentage of 10% was assigned.

Seven departments reported production of their own immobilisation equipment; custom head rests and hand moulds were the most common, occurring in two departments each. Other items included headrest spacers, magnetic resonance imaging simulation locator bar attachments, Deep Inspiration Breath Hold (DIBH) positioning devices, neck wedges, Vacbag/2‐part‐foam locators, magnetic resonance linac aids and a mouth bite valve. Of the quality assurance equipment printed, four departments made chamber holders, two departments made phantom holders and two departments made phantoms (one with fiducials). Other items included frames, nuts, bolts, levers, a half value layer insert holder, Varian Real‐time Position Management blocks, DIBH stands, thermoluminescent dosimeter/film holders for total body irradiation, six‐degrees of freedom phantom positioning device and an Ion chamber tube with a custom phantom.

The single department that reported use of their printer to make electron inserts did not make the physical electron insert with their 3D printer. Electron patients at this department underwent a 3D surface scan of the treatment area, which was then converted into a physical 3D print and CT scanned into the treatment planning system (TPS) as the patient surrogate.

### Characterisation of filament properties in treatment planning systems

**Figure 4:**
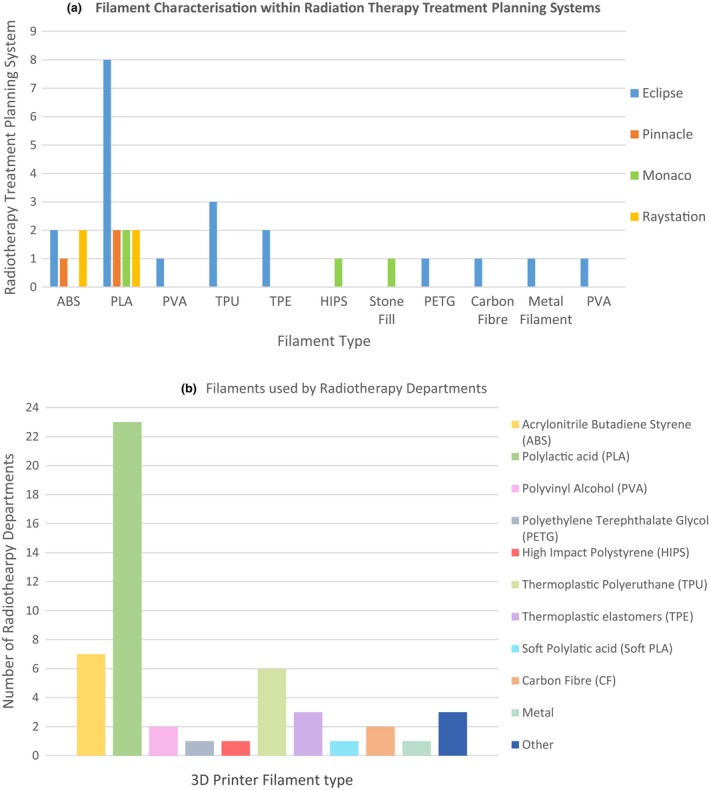
Filament characterisation and use in radiotherapy. (a) Filament characterisation within radiation therapy treatment planning systems (b) Filaments used by radiotherapy departments.

Twelve departments had characterised the filament properties within their TPS as shown in Figure 4A. Only one department reported using more than one TPS; Raystation (Raysearch Laboratories AB, Stockholm, Sweden), Pinnacle (Koninklijke Philips N.V., Amsterdam, Netherlands) and Eclipse (Varian Medical Systems, Palo Alto, California, USA) but did not specify if their ABS and PLA printing filaments had been characterised across all three systems. It is also unclear if all filaments listed had been characterised within the TPS, as the survey questions did not differentiate between materials used clinically within a treatment beam or manufactured as support or quality assurance equipment. Figure 4B provides a comprehensive list of the type of filament in use, with PLA the most popular, used by 23 departments.

### Location of 3D printer

**Figure 5:**
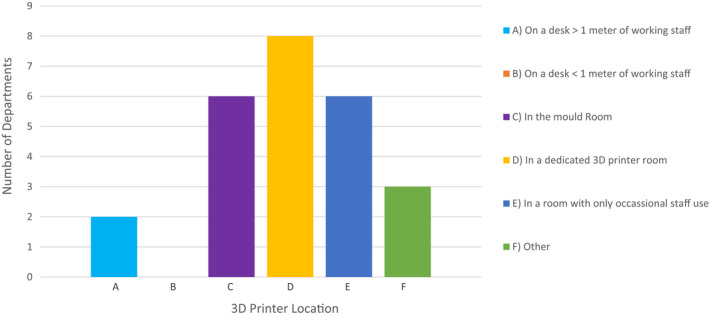
3D printer storage.

Only six departments reported having ventilation requirements for 3D printers (Fig. 2). Two of these departments stored their printer in a dedicated 3D printer room, two departments stored the printer in mould rooms, one department used an old bunker, and the last department stored the printer under a fume hood in a room and was only used occasionally. Figure 5 details where all twenty‐five 3D printers were stored, including those detailed above.

### Software

**Figure 6:**
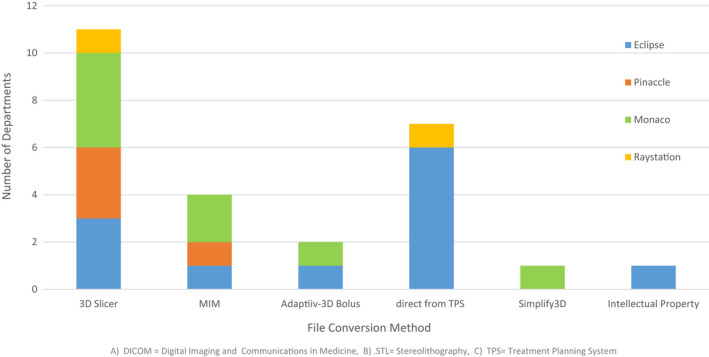
Software used for DICOM^A^ to .STL^B^ file conversion and TPS^C^ in use.

Standard Tessellation Language or stereolithography (.stl) file format was the most common file format used in 3D printing. The most popular method for converting Digital Imaging and Communications in Medicine (DICOM) files into a .stl file format was the use of 3D Slicer (https://www.slicer.org/), a free open‐source software platform that allows medical image processing and 3D visualisation.^7^ Figure 6 lists DICOM to .stl file conversion methods based on departments TPS.

Nine departments reported using one or more methods of smoothing software on their 3D structure sets. Meshmixer (Autodesk, Inc. San Rafael, California, USA) was the most popular smoothing software, used by four departments, followed by Meshlab (https://www.meshlab.net, National Research Council, Pisa, Italy), Adaptiiv (Adaptiiv Medical Technologies, Halifax, Canada) and Eclipse TPS by two departments each. Cura (Ultimaker, Gelderland, The Netherlands) and Raystation TPS were also employed for smoothing by a single department, respectively.

### Liked and disliked 3D printer features

Survey respondents were asked to list the features of their printer they liked or disliked the most. The responses per printer model can be found in Table 1. Of the 50 answers given for most liked features, the top survey response was a simple user interface (28%), followed equally by a heated print bed and reasonable size build volume (16% each). A dual extruder was the next popular response (12%).

**Table 1:** Most liked and disliked 3D printer features.

LIKED Printer Features	Creality	Cubicon	Cultivate	Lultzbot	Kühling & Kühling	Vivedino	Seemecnc	Upbox	Zortrax	AON‐M2	Raise 3D	Ultimaker
Simple to use	1						1	1	1	1	3	6
Cheap price point	1					1						
Good quality prints	1											
Heated bed		2			1		1		1		1	2
Heated chamber		2										
Filtration system		1			1							
Reasonable build volume		1	1		1				1		2	2
Easy part removal		1										1
Robust				1								1
Dual head/extrusion					1	1					2	2
Extruder temperature range											1	1
Filament options											1	
Easy repair												1
Custom settings												1
Cost effective												1
Fast prints												1
DISLIKED Printer Features												
Z‐axis instability for large prints	1											
Lack of filament sensor	1											1
No camera for monitoring		1								1		1
Issues with support		1										
Issues with replacement parts		1										
Limitation of filament supplier		1										
Small print bed		1						1			1	2
Single extruder		1										2
Sensor issues			1									
Long print times				1								
No resume print on interrupt option				1								
Filament getting stuck					1							
Not enclosed to control ambient conditions						1	1					
Nozzle temperature issues							1				1	
Filament loading position									1			
SD Card/USB file transfer												3
No inbuilt ventilation												1

Of the 30 answers given for most disliked features, the top survey response was a small print bed (16.7%), followed equally by lack of an onboard camera for monitoring printing in real time, a single extruder and SD card or USB stick file transfer (10% each).

### Outsourced 3D printer products

Three public and thirty‐one private Australian departments source 3D printed products from private providers. There was no outsourcing of 3D products in New Zealand. Two of three public departments that used outsourced 3D products had characterised the 3D filament properties in their TPS and performed geometry checks, density checks, in vivo dosimetry and CT fusion of the provided 3D products. All private departments performed planning simulation with 3D printed products in place. Pinnacle is the TPS used for the three public departments, while the single representative who responded on behalf of all 31 private departments noted various TPS across the company.

Thirty‐four departments reported the purchase of privately sourced custom bolus. This bolus was for both electron and photon treatments, with one department not specifying modality.

For the thirty‐two departments that outsourced electron inserts, standard shape and size inserts were commissioned during implementation into each department. Output factors were obtained for each custom electron insert, with a relevant aperture check. One public department reported purchasing ‘physics related quality assurance equipment/accessories, with physics assessment quality assurance checks’. One public department had also reported purchasing moulds for complex wax/silicone bolus construction. These moulds were printed with polyvinyl alcohol (PVA) and thermoplastic polyurethane (TPU).

Thirty‐three departments ordered 3D products made from TPU and two departments ordered PLA products. PVA moulds for wax, silicone and tungsten impregnated with resin pressed into an electron mould made up the other materials sourced privately. Only one department owned a printer but outsourced 3D printed products due to changes in management. No immobilisation equipment was purchased privately. All departments reported being happy with the quality of 3D products and accessories purchased to date.

## Discussion

This cross‐sectional study was successful in establishing a baseline for Australian and New Zealand 3D printer ownership and 3D printed product use within RT and will be helpful for departments investigating the purchase of a 3D printer.

### Department‐owned 3D printers

Based on responses received from the survey, there appeared to be no standard process for 3D printing protocols and usage across Australia and New Zealand. Variations exist across most aspects, from storage of device, infill percentages and filament material. One department stated that they disliked the fact their model of printer could not print flexible filament, conversely two other departments with the same model of printer reported producing successful prints with flexible filaments. This demonstrates that expertise in 3D printer use requires time to understand the functionality of the 3D printer and some testing of printer settings to perfect the use of a wide variety of filaments.

Of the four departments that report owning more than one 3D printer, three departments reported making bolus for both electron and photon treatments. The fourth department did not identify a use for their four or more printers, but did identify smoothing software, TPS, and infill percentage that they use. There was no correlation between the increased number of printers used, and an increase in applications of use. Future investigations would benefit from defining all uses for the printer. An additional question on the total number of 3D printed products produced by each department, and number of hours per week the printer is in use would also be beneficial. This would help establish how well integrated 3D printers are in department workflow and if 3D printers are used routinely or reserved for use in unique circumstances.

Rooney et al found in 2020 that globally 3D printed bolus use was divided into 71% photons, 24% electrons, and 6% protons.^1^ This compared to 56% photons (14 departments), and 28% electrons (7 departments) established by this survey. Proton therapy is not available in Australia or New Zealand. The question regarding infill percentage (question 20 and 21) was an open‐ended survey response that did not specify the end use for the item being printed but did allow a free text repose to define use (Fig. 3). The intention of this question was to ascertain common bolus and immobilisation equipment infill percentages; however, only one survey respondent annotated ‘bolus’ in the response for photon infill percentage. Future investigations would benefit by clarifying end use of the 3D printed product and its percentage infill.

Survey results show 14 departments were 3D printing bolus, 7 departments were producing immobilisation devices, and 10 departments were producing quality assurance equipment/phantoms. This indicates that 3D printing has the capacity to replace many of the functions of a traditional mould room such as patient casts, immobilisation equipment and bolus, and would allow for more flexibility in the tailoring of dosimetry to patients' needs. With the use of biodegradable materials in the printing process, 3D printing may also prove more environmentally sustainable than established mould rooms following traditional manufacturing techniques with toxic metals and non‐biodegradable materials.

The long‐term effects of working in a room with a 3D printer have not been investigated, although the low percentage of centres which place their printers in a designated ventilation space (24%) may indicate that many do not think there is a health risk to long‐term inhalation of fumes.^8^ Of the 25 departments who owned a 3D printer, only one department identified lack of inbuilt ventilation as a disliked printer feature.

Question 19 asked respondents about characterising filament properties. The literature has established that ‘It is not acceptable to assume that 3D printed materials are water equivalent, or that the TPS will accurately manage these materials using the CT calibration curves’.^2^ Of departments who have implemented a 3D printing program, only 52% have characterised the filament properties within their TPS. Of the 14 departments currently 3D printing bolus, 11 have characterised the filament properties within their TPS. It is unknown how the remaining 3 departments verify the characteristics of the bolus they produce. As electron density variation within bolus can have significant impacts on dosimetry, it is vital to be confident that 3D printed bolus exhibits the expected characteristics. Future investigations would benefit from exploring what guidelines and clinical protocols departments have put in place to verify the product they have produced is exhibiting all required characteristics.

In February 2021, the Australian Therapeutic Goods Administration (ATGA) released guidelines specific to the use of 3D printed products.^9^ Application of the guidelines within the Radiotherapy environment will vary depending on the intended outcome of the 3D printed product. Departments who established clinical guidelines to produce 3D products for a set purpose, need to apply to the ATGA for approval to be registered. However, those departments that produce one off custom designed 3D products, with a narrow focus of use that cannot be applied to any other clinical situation, and at a rate of less than 5 items per year, do not require registration.

All forms of bolus used within RT are considered patient‐matched medical devices under ATGA guidelines. There are a total of 11 entries for RT bolus on the ATGA register (as of Feburary 2022), two specific to individual departments and nine by commercial suppliers. Of the two departments that have registered the use of bolus, only one includes 3D printed material.

MEDSAFE, the New Zealand Medicines and Medical Devices Safety Authority is the New Zealand equivalent of the ATGA, and their database of medical devices is WAND (Web‐Assisted Notification of Devices). There is no public access to the WAND database, and no mandatory requirement for medical devices to be assessed in terms of quality, safety, efficacy or performance by a medical regulator within New Zealand. All 3D printed products that comply with WAND regulations are exempt from notification under Schedule 1 of the Medicines (Database of Medical Devices) Regulations 2003 act, where ‘medical devices manufactured to the specifications of a healthcare practitioner for a particular patient or are supplied to a healthcare professional for use in relation to a particular patient of the healthcare professional’ are exempt from notification.^10^

### Outsourced supply of 3D printed product

As commercial supply of 3D printed products for medical use is regulated through ATGA, the register forms a viable reference for departments in Australia to locate companies who have registered their 3D printed products.

Thirty‐four departments reported positive results from outsourcing their 3D products and were unable to list any disadvantages. Future versions of this survey would benefit from including questions specific to department workflow and the timeline surrounding product design, manufacture and arrival within the department. As no disadvantages were listed for outsourcing 3D printed products, it implies that departments accept any delays associated with redesign, manufacture and delivery.

For departments outsourcing electron inserts, it is unknown how many patients were treated with electrons per year, and if this was a determining factor for departments to transition to external supply. Traditional manufacture of electron inserts requires mould room training, a ventilation space and personal protective equipment. If the alloy used to make the insert contains lead (e.g. cerrobend), then staff safety must be taken into account in the daily handling of the insert. It is possible that a review of safe work practices, awareness of a new alternative to inhouse manufacture, or cost benefit analysis have led to the thirty‐two departments outsourcing electron inserts. Future investigations would benefit from examining the number of electron treatments per year in these departments and changes to staff resourcing.

### Limitations

Responses for ‘liked’ and ‘disliked’ 3D printer features were likely influenced by the survey question examples. A more thorough response from departments may have been achieved by identifying every possible feature of a 3D printer, with an option to choose ‘like’ or ‘dislike’.

## Conclusion

With almost a quarter of Australian and New Zealand RT departments now in ownership of a 3D printer (22.3%), 3D printing within the clinical and quality assurance settings has been shown to be widespread. With a number of uses such as in immobilisation equipment, phantoms, bolus and equipment holders and positioners, there is a vast array of scope for the further integration of 3D printed products in many more departments.

Even without having a 3D printer, 34 departments employ external services for the creation of 3D printed products, further supporting the demand that is now in place. As 3D printing becomes more affordable and easier to use, its integration into RT will likely see an increase in the number of departments using 3D printed products or owning a 3D printer in the future. The result of this will be improved patient care through tailored dosimetry and personalised immobilisation aids.

## Conflicts of Interest

We have no known conflicts of interest to disclose.

## Supporting information


**Appendix** S1: Supporting information

## Appendix A Radiation therapy department locations

**Table:**

Location	Government and Other Websites
Australian Departments	https://www1.health.gov.au/internet/main/publishing.nsf/Content/health‐roi‐radiother‐index.htm
	https://www.targetingcancer.com.au/treatment‐centres/
	http://sbcrowe.net/australian‐centres/
	https://www.aihw.gov.au/reports/radiotherapy/radiotherapy‐in‐australia‐2018‐19/contents/radiotherapy‐activity‐and‐collection‐participation
New Zealand Departments	https://www.targetingcancer.com.au/treatment‐centres/
	http://sbcrowe.net/new‐zealand‐centres/
	https://www.health.govt.nz/system/files/documents/publications/national‐radiation‐oncology‐plan‐may17.pdf

## References

[1] Rooney MK , Rosenberg DM , Braunstein S , et al. Three‐dimensional printing in radiation oncology: A systematic review of the literature. J Appl Clin Med Phys 2020; 21: 15–26. 10.1002/acm2.12907 PMC7484837 32459059

[2] Craft DF , Kry SF , Balter P , Salehpour M , Woodward W , Howell RM . Material matters: Analysis of density uncertainty in 3D printing and its consequences for radiation oncology. Med Phys 2018; 45: 1614–21. 29493803 10.1002/mp.12839

[3] Solc J , Vrba T , Burianova L . Tissue‐equivalence of 3D‐printed plastics for medical phantoms in radiology. J Instrum 2018; 13: P09018.

[4] Biltekin F , Yazici G , Ozyigit G . Characterization of 3D‐printed bolus produced at different printing parameters. Med Dosim 2020; 46: 157–63. 33172711 10.1016/j.meddos.2020.10.005

[5] Van der Walt M , Crabtree T , Albantow C . PLA as a suitable 3D printing thermoplastic for use in external beam radiotherapy. Australas Phys Eng Sci Med 2019; 42: 1165–76. 31728939 10.1007/s13246-019-00818-6

[6] Fujimoto K , Shiinoki T , Yuasa Y , Hanazawa H , Shibuya K . Efficacy of patient‐specific bolus created using three‐dimensional printing technique in photon radiotherapy. Phys Medica 2017; 38: 1–9. 10.1016/j.ejmp.2017.04.023 28610688

[7] Kamio T , Suzuki M , Asaumi R , Kawai T . DICOM segmentation and STL creation for 3D printing: a process and software package comparison for osseous anatomy. 3D Print Med 2020; 6: 1–12. doi: 10.1186/s41205-020-00069-2 32737703 PMC7393875

[8] Stephens B , Azimi P , El Orch Z , Ramos T . Ultrafine particle emissions from desktop printers. Atmos Environ 2013; 79: 334–9.

[9] Personalised medical devices (including 3D‐printed devices). https://www.tga.gov.au/resources/resource/guidance/personalised‐medical‐devices‐including‐3d‐printed‐devices

[10] Medicines (Database of Medical Devices) Regulations 2003 (SR 2003/325) (as at 07 August 2020) Contents – New Zealand Legislation

