# Supplementary material for: Three‐dimensional printer use in the Australian and New Zealand radiation therapy setting
Source: J Med Radiat Sci. 2022 Sep 12;69(4):492–501. doi: 10.1002/jmrs.613 (PMC9714517; doi:10.1002/jmrs.613)
Supplement: Supplementary file 1 — Appendix S1: Supporting information [file JMRS-69-492-s001.docx]

Supporting Information

| *Survey Questions* | |
| --- | --- |
| Q1 | Department (name): ……………………………………………………………………………………………………………… |
| Q2 | Contact Details (name, email, phone number): ….………………………………………………………………… |
| Q3 | Do you have a 3D printer in the department? ….……………YES ………………NO…. (jump to Q31) |
| Q4 | How many 3D printers do you have? …………………………………………............................................ |
| Q5 | What is the make and Model of your 3D printer? |
| Q6 | Do you use the 3D printer to make a mould for bolus? ….....….YES ..….….…NO……(jump to Q8) |
| Q7 | What material do you use in the 3D printed mould to create your bolus? |
| Q8 | Do you use the 3D printer to make Bolus? ………………………YES………………….NO…………..…… |
| Q9 | Is this bolus for use with Electrons? …………………………YES………………….NO…………………N/A…….. |
| Q10 | Is this bolus for use with Photons? ………….………….……YES………………….NO………..……… N/A …… |
| Q11 | Do you use the 3D printer to make Immobilization equipment? …..YES .….NO….(jump to Q13) |
| Q12 | What immobilization equipment have you made? |
| Q13 | Do you use the printer to make QA phantoms? ………..……….…….…YES………….…….NO…..………. |
| Q14 | Do you use the 3D printer to make QA equipment? …..……….….YES……....NO…..(jump to Q16) |
| Q15 | What QA equipment have you made? |
| Q16 | Do you use the 3D printer to make electron inserts? …..….…YES….…..….NO…..… (jump to Q18) |
| Q17 | How do you use the 3D printer to make electron inserts? |
| Q18 | What 3D Filament do you print with?  ABS, PLA, PVA, HIPS, PET, PETT, NYLON, TPE, Carbon Fiber,  *Flexible Filaments:* TPU, TPC, TPA, Soft PLA,  *Metal Filament:* red copper, brass, bronze, aluminum, copper  Other:………………… |
| Q19 | Have you characterized the filament properties in your treatment planning system? YES / NO |
| Q20 | What treatment planning system do you use? |
| Q21 | What infill % do you print with for Photon treatments? |
| Q22 | What infill % do you print with for Electron treatments? |
| Q23 | Do you have a ventilation requirement for 3D printer use? …………….…YES…………...NO…….…… |
| Q24 | Where do you store your 3D printer? *(select more than one if appropriate)*   - On a desk within >1m of working staff - On a desk within <1m of working staff - In the mould room - In a dedicated 3D printer room - In a room with only occasional staff use |
| Q25 | How do you convert your DICOM structure to a .STL file? |
| Q26 | Do you apply smoothing software to your DICOM structure? ………….…YES……….....NO…….…… |
| Q27 | What smoothing software do you use? |
| Q28 | What features of your 3D printer do you ***like*** the most? |
| Q29 | What features of your 3D printer do you ***dislike*** the most? |
| Q30 | Do you employ the services of a private company to supply 3D printed products to your  department? ………..…………...….…YES……..........…...….NO……………..(end of survey, thank-you) |
| Q31 | Have you characterized the filament properties of your supplied 3D printed products in your  treatment planning system? ………..…………….…YES……..……………...….NO…………..…………………. |
| Q32 | What treatment planning system do you use? |
| Q33 | Have you purchased patient specific 3D printed bolus? …………YES.…..…..NO…..(jump to Q37) |
| Q34 | Was this bolus for electron treatments? …………………….….…YES………..………….....NO………….…… |
| Q35 | Was this bolus for photon treatments? ………………………….…YES………………….…...NO………….…… |
| Q36 | What QA do you perform on this bolus? |
| Q37 | Have you employed the use of a 3D printing company to custom make patient specific  immobilization equipment? ………..……………….…YES……..………………..….NO……(jump to Q39)….. |
| Q38 | What immobilization equipment has been made? |
| Q39 | Have you employed the use of a 3D printing company to custom make QA equipment and  accessories? ………..…………..….…YES……..……..………...….NO…………..…(jump to Q41)…………… |
| Q40 | What QA equipment and accessories have been made? |
| Q41 | Have you purchased patient specific electron inserts? …..….…YES….…..….NO……(jump to Q43) |
| Q42 | What QA do you undertake on purchased 3D printed electron inserts? |
| Q43 | Are you happy with the quality of the 3D printed products that you have purchased for  patient specific purposes? ……….…YES… (jump to Q45)……..……………..….NO……………………… |
| Q44 | Why were you unhappy with the quality of the 3D printed product? |
| Q45 | Are you happy with the quality of the 3D printed accessories you have purchased for use in  your Radiotherapy department?  ………N/A……(jump to Q47)  ......…YES……(Jump to Q47)  …...….NO…… |
| Q46 | Explain why you were unhappy with the quality of 3D printed accessories you have purchased. |
| Q47 | What material is used to produce the 3D printed products you purchase (i.e., PLA, ABS etc.)? |
| Thank-you for your time completing this survey. | |
